# The impact of serum uric acid on psoriasis: NHANES 2005–2014 and Mendelian randomization

**DOI:** 10.3389/fgene.2024.1334781

**Published:** 2024-05-09

**Authors:** Minghui Hu, Yangyang Wang, Wenwu Xu, Juan Bai, Xingming Tang

**Affiliations:** ^1^ Department of Orthopedics, DongGuan SongShan Lake Tungwah Hospital, Dongguan, Guangdong, China; ^2^ Department of Internal Medicine, DongGuan SongShan Lake Tungwah Hospital, Dongguan, Guangdong, China; ^3^ Department of Orthopedics, Dongguan Tungwah Hospital, Dongguan, Guangdong, China

**Keywords:** uric acid, psoriasis, NHANES, cross-sectional study, Mendelian randomization, causality

## Abstract

**Background:**

Psoriasis is a chronic systemic inflammatory disease, and hyperuricemia is a common comorbidity in patients with psoriasis. However, the exact relationship between uric acid levels and psoriasis remains unclear. This study aimed to explore the association between uric acid levels and psoriasis.

**Methods:**

Observational study participant data (≥16 years, n = 23,489) from NHANES 2003–2014. We conducted analyses using a weighted multiple logistic regression model. Genetic data sets for uric acid levels and psoriasis were obtained from the IEU database. We selected genetically independent loci closely associated with serum uric acid levels as instrumental variables and performed Mendelian randomization analyses using five complementary methods: inverse variance weighting (IVW), MR-Egger, weighted median, simple mode, and weighted mode.

**Results:**

After adjusting for other covariates, the results revealed no significant association between serum uric acid levels and psoriasis (b = 0.999, 95% CI: 0.998, 1.001, *p* = 0.275). Subgroup analyses stratified by gender and ethnicity showed no significant association between sUA and psoriasis in any of the subgroups. Furthermore, the MR analysis involved the selection of 227 SNPs that were associated with both sUA and psoriasis. IVW results demonstrated no causal relationship between sUA and psoriasis (OR = 0.282, 95% CI: -0.094–0.657, *p* = 0.142).

**Conclusion:**

Our study suggests that uric acid levels are not significantly causally related to psoriasis. This finding provides valuable insights for the treatment and prevention of psoriasis, indicating that merely reducing uric acid levels may not be an effective strategy to reduce the risk of psoriasis onset.

## 1 Introduction

Psoriasis, a common chronic inflammatory skin disease characterized by erythematous and scaly plaques. However, its impact extends beyond the skin, affecting nails, joints, and potentially leading to various comorbidities (Uppala et al., 2021). The global prevalence of psoriasis varies between 0.09% and 5.10% (Michalek et al., 2017), significantly impacting the quality of life for those affected and imposing a substantial burden on individuals and society at large (Griffiths et al., 2021). The pathogenesis of psoriasis is multifaceted, involving genetic, immunological dysregulation, and environmental factors (Jensen and Skov, 2016). As the prevalence of inflammatory arthritis, cardiovascular diseases, and metabolic syndrome continues to rise, the management of psoriasis becomes notably intricate (Martinez-Moreno et al., 2021; Wu et al., 2022). Current treatment approaches encompass various interventions, with mild cases often treated using topical medications (Armstrong and Read, 2020), while moderate to severe cases necessitate the use of biologics targeting TNF-α, p40IL-12/23, IL-17, p19IL-23, and even oral phosphodiesterase-4 inhibitors (Merola et al., 2015). Nevertheless, these treatments remain incapable of curing the disease. Hence, investigating its epidemiological characteristics and risk factors is crucial. Understanding the etiology could facilitate the development of safe and effective therapeutic strategies for reducing disease relapse and extending remission periods. Presently, alongside the known risk factors, research suggests a significant correlation between elevated serum uric acid (SUA) levels and psoriasis (Zhang et al., 2021). SUA is a metabolite of purine nucleotides derived from both dietary sources and internal nucleic acid metabolism. Under normal conditions, SUA levels range from 1.5 to 6 mg per deciliter for females and 2.5–7 mg per deciliter for males (Chen et al., 2020). Although moderate SUA levels are recognized as beneficial antioxidants, hyperuricemia or excessive SUA levels have been confirmed as independent risk factors for various diseases, including metabolic syndrome, diabetes, hypertension, cardiovascular events, and kidney disorders (Chen et al., 2022; Nishizawa et al., 2022; Jiang et al., 2023). However, the precise relationship between uric acid levels and psoriasis remains unclear, and there is a scarcity of randomized controlled trial (RCT) studies investigating the impact of uric acid on psoriasis, with only a limited number of experimental and epidemiological studies having explored this association. To delve deeper into the relationship between uric acid and psoriasis, this study employs a combined approach utilizing data from NHANES and Mendelian randomization (MR) analysis.

NHANES is a nationwide research survey conducted biennially in the United States, designed to assess the health and nutrition status of American adults and children. This comprehensive program utilizes a multi-stage, stratified survey design and collects diverse information through interviews, physical examinations, and laboratory tests (Ahluwalia et al., 2016). In this study, we employ NHANES data to investigate the association between uric acid levels and psoriasis. While NHANES offers a wealth of health and nutrition data, its cross-sectional design limits the ability to establish causal relationships. Self-reported data may also be influenced by memory biases and social desirability. To address these limitations, MR methodology is employed. MR serves as a robust tool for discerning potential causal relationships between various exposure factors and clinical outcomes. MR functions similarly to RCTs, as genetic variation is randomly assigned during gametogenesis, akin to the random allocation of interventions in RCTs (Emdin et al., 2017; Sanderson et al., 2022). MR analysis employs single nucleotide polymorphisms (SNPs) as instrumental variables (IVs) to infer potential causal relationships between exposure factors and disease outcomes. Given that genetic variants are randomly assigned at conception and are impervious to environmental or lifestyle confounders, MR methodology is less susceptible to confounding factors (Emdin et al., 2017). The complementary strengths of NHANES and MR make them ideal tools for investigating complex health issues in a rigorous and systematic manner.

Therefore, this research aims to investigate the relationship between uric acid levels and psoriasis by integrating two analytical approaches: NHANES cross-sectional study and MR methodology. Utilizing NHANES data provides extensive clinical information, while MR methodology, leveraging genetic variation as instrumental variables, enables the control of confounding factors. This combined approach enhances the precision of our assessment regarding the potential causal relationship between uric acid levels and psoriasis onset.

## 2 Methods

### 2.1 Data source

To begin, we conducted an epidemiological analysis utilizing data from the NHANES (https://www.cdc.gov/nchs/nhanes/index.htm). NHANES is a nationally authorized survey project by the National Center for Health Statistics (NCHS), designed to assess the health and nutritional status of the American population. NHANES employs both self-reported and objectively measured data. Self-reported data are obtained through structured interviews conducted in participants’ homes, while objective measurements are collected during physical examinations and laboratory tests conducted in mobile examination centers. All NHANES participants provided written informed consent before their participation. As this is a secondary analysis, no additional institutional review board approval was required.

#### 2.1.1 Variable acquisition

**FIGURE 1:**
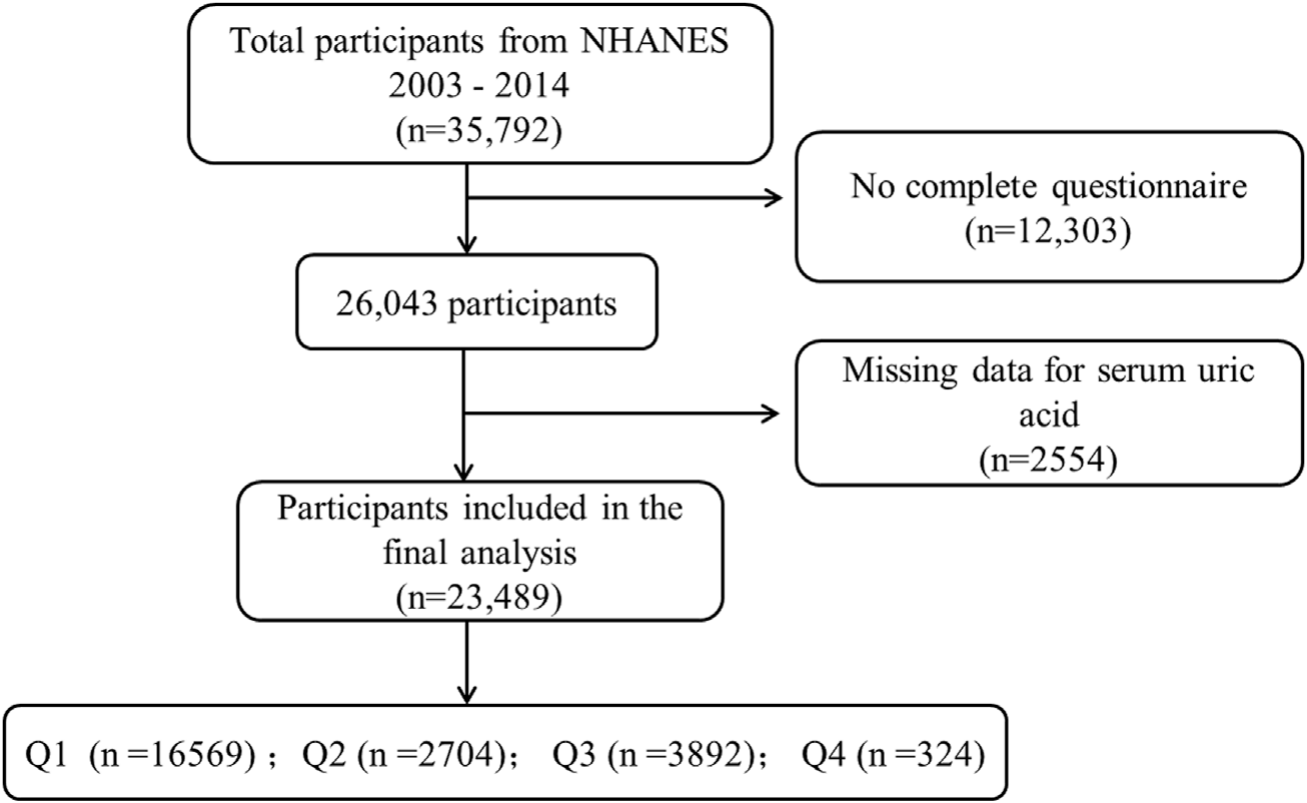
NHANES data screening flow chart. * Serum uric acid levels are divided into quartiles Q1, Q2, Q3 and Q4 from low to high.

In this cross-sectional study, our research focused on the years 2005–2014 as this timeframe was chosen due to the availability of psoriasis data exclusively within this period of NHANES data collection. A total of 35,792 participants aged 16 and above were included in our study (Figure 1). Psoriasis status was ascertained based on responses to the question, “Have you ever been told by a healthcare provider that you have psoriasis?” Among the respondents, a total of 23,489 individuals with psoriasis were included in our analysis after completing the psoriasis questionnaire.

SUA is a product of uricase oxidation, resulting in uric acid and hydrogen peroxide (Ma et al., 2014). SUA data were analyzed as continuous variables, and high uric acid levels were defined according to established diagnostic criteria as SUA levels exceeding 416 μmol/L for males and 357 μmol/L for females (Gao et al., 2023).

#### 2.1.2 Covariate acquisition

Covariates were assessed based on previously described methods and clinical practices (Ellis et al., 2019; Xu et al., 2023), encompassing a range of potential factors, including gender, age, ethnicity, education level, body mass index (BMI), smoking status, alcohol consumption, hypertension, and diabetes. Additionally, data related to psoriasis were included for analysis following the selection criteria from relevant studies.

#### 2.1.3 Statistical analysis

All statistical analyses were conducted using R software (version 4.3.1). Categorical variables were presented as frequencies or percentages, while continuous variables were reported as mean ± standard deviation. Serum uric acid levels were divided into quartiles (Q1, Q2, Q3, and Q4) based on ascending order. After adjusting for potential confounding factors, a weighted multivariate regression analysis was employed to estimate the independent relationship between SUA and psoriasis. All analyses took into account the complex multi-stage sampling design of NHANES and were weighted using the provided sampling weights (He et al., 2019). The statistical significance level was set at a *p*-value less than 0.05.

### 2.2 Mendelian randomization

**FIGURE 2:**
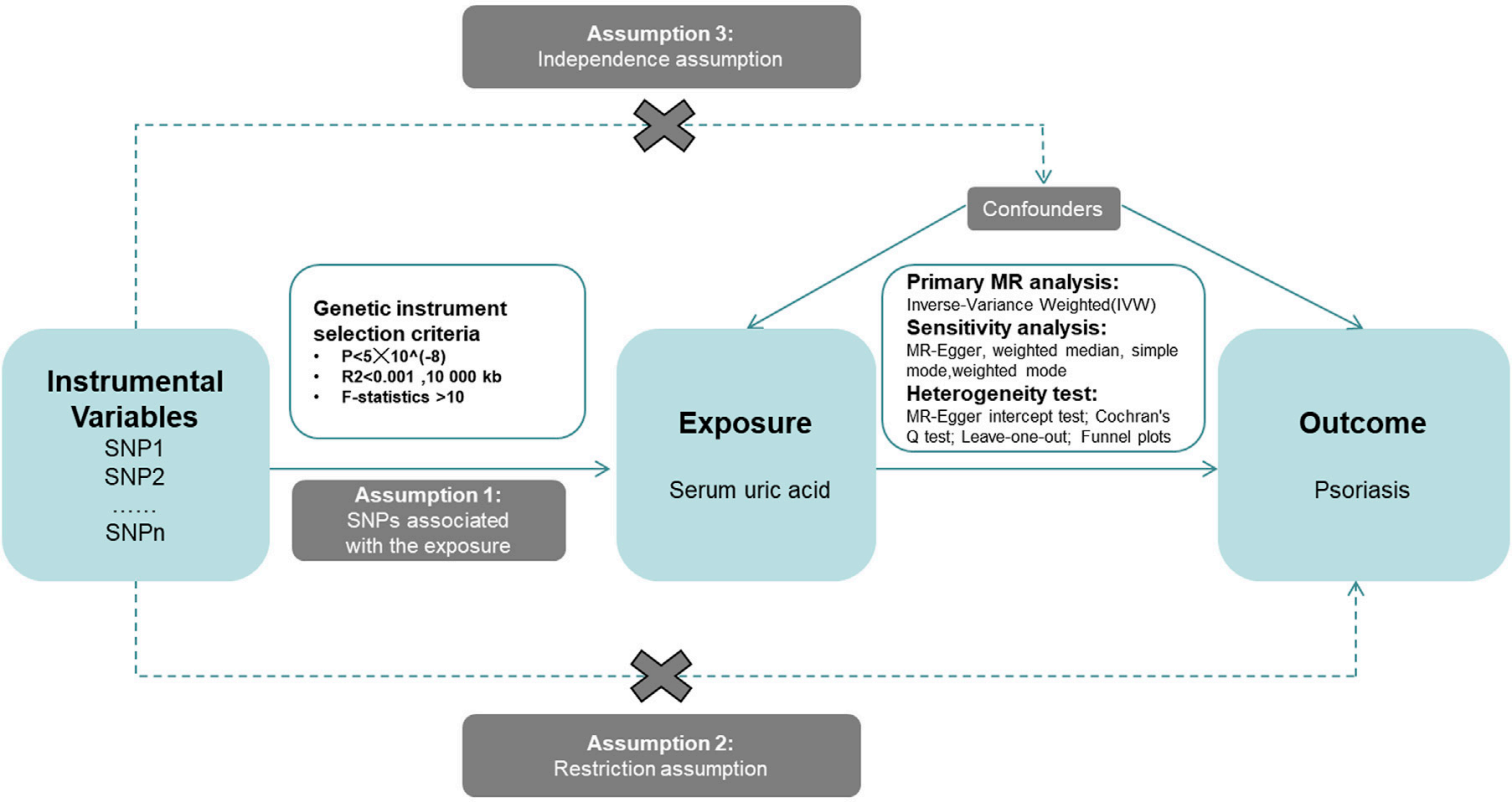
Research framework diagram of Mendelian randomization study revealing the causal relationship between serum uric acid and psoriasis.

This study aims to explore the causal relationship between Serum uric acid levels and psoriasis using an MR design. First, we acquired data related to SUA and psoriasis, which served as the exposure factor and the disease outcome, respectively. Subsequently, we employed five complementary MR methods to analyze the relationship between these two variables. These methods included Inverse-Variance Weighted (IVW), MR-Egger, Simple Mode, Weighted Mode, and Weighted Median. The study frame chart is presented in Figure 2.

#### 2.2.1 Data sources

The data for this study are based on publicly available summary statistics from Genome-Wide Association Studies (GWAS). As we utilized pre-published, publicly accessible databases, no additional ethical approval was required. Uric acid data, serving as the exposure variable, were obtained from a GWAS study encompassing 129,405 individuals (Kanai et al., 2018), involving 12,499,459 SNP loci. Data for the disease outcome, Psoriasis, were derived from Sakaue’s GWAS Mate analysis (Sakaue et al., 2021), involving 172,495 individuals and 12,453,837 SNP loci. These databases were selected based on their inclusion of large-scale sample data, enhancing the statistical power of MR analysis. Comprehensive data for all relevant analyses can be publicly accessed on the database website (https://gwas.mrcieu.ac.uk). Notably, the data in these GWAS studies are drawn from European populations, as summarized in Supplementary Table S1.

#### 2.2.2 Selection of genetic variants

In this study, we adopted a series of stringent steps to ensure the reliability of the selected IVs and to meet the requirements for conducting Mendelian randomization. The following steps outline our IV selection process: Initially, we chose single nucleotide polymorphisms (SNPs) significantly associated (P < 5E-8) with uric acid, our exposure variable. SNPs in linkage disequilibrium (LD) with selected IVs were excluded to ensure independence. PhenoScanner (Kamat et al., 2019) aided in identifying and excluding SNPs correlated (P < 5E-8) with potential confounding factors. IVs’ strength was assessed using the F-statistic (Brion et al., 2013), requiring values exceeding 10. IVs related to uric acid were extracted from comprehensive data involving psoriasis. Proxy SNPs (R2 > 0.9) were found if direct extraction wasn't feasible. Data consistency checks ensured uniform IVs for MR analysis. Through these steps, we obtained a reliable set of IVs to be used in the subsequent MR analysis, enabling the investigation of the causal relationship between uric acid and psoriasis.

#### 2.2.3 Statistical analysis

This study employed five different MR methods to estimate the causal relationship between uric acid and psoriasis. These methods include IVW, MR-Egger regression, Weighted Median, Simple Mode, and Weighted Mode. The IVW method functioned as the primary MR analytical tool. This approach ensures a thorough examination of the potential causal relationship between uric acid levels and psoriasis while minimizing biases. Results with *p*-values less than 0.05 were considered statistically significant.

#### 2.2.4 Sensitivity analysis

In this study, sensitivity analyses were conducted to assess the robustness and reliability of our MR results. The inclusion of these analyses is crucial to validate the conclusions drawn from MR analysis and ensure they are not driven by any single genetic variant or assumption. We employed several sensitivity analysis methods, for estimates of significance, we conducted MR-Egger intercept tests and leave-one-out analyses to further evaluate horizontal pleiotropy. Cochran’s Q-test was employed to determine heterogeneity. Funnel plots were used to assess potential directional pleiotropy, similar to their use in meta-analyses to evaluate publication bias. All analyses were conducted using the TwoSampleMR package (version 0.4.25) and the MRPRESSO package (version 3.6.1) in R. The significance level was set at α = 0.05.

## 3 Results

### 3.1 Results of the NHANES data analysis

After screening, a total of 23,489 study subjects were included in this research. The characteristics of the study subjects were further stratified based on SUA quartiles. Among them, 11,360 (48.36%) were male, 10,193 (43.39%) were White, 6,716 (28.59%) had a college education or higher, 4,904 (20.89%) were smokers, 5,647 (87.68%) were non-drinkers, 6,956 (29.61%) had hypertension, 2,254 (9.59%) had diabetes, and 2.62% were psoriasis patients. Significant differences were observed among SUA quartiles (Q1-Q4) in gender, age, race/ethnicity, BMI, smoking and drinking behaviors, hypertension, and diabetes (*p* < 0.001), but not in education level and psoriasis (*p* > 0.05) (Table 1).

**TABLE 1:** Characteristics of participants.

Variable	Total (n = 23,489)	Q1 (n = 16,569)	Q2 (n = 2,704)	Q3 (n = 3,892)	Q4 (n = 324)	*p*-value
psoriasis						>0.05
Yes	616	412	79	112	13	
No	22,873	16,157	2,625	3,780	311	
Gender						<0.001
Female	12,129	10,483	717	849	80	
Male	11,360	6,086	1987	3,043	244	
Age (mean ± ME)	46.76 ± 0.29	46.23 ± 0.28	47.20 ± 0.45	48.3 ± 0.44	52.46 ± 1.20	<0.001
Race/ethnicity						<0.001
white	10,193	7,027	1,215	1818	133	
black	5,027	3,414	572	937	104	
Other Hispanic	1954	1,491	199	238	26	
Other	6,315	4,637	718	899	61	
Education						>0.05
9-11th Grade (Includes 12th grade with no diploma)	3,279	2,315	381	530	53	
College Graduate or above	5,256	3,793	575	837	51	
High School Grad/GED or Equivalent	5,209	3,620	593	920	76	
Less Than 9th Grade	2,263	1,599	258	373	33	
Some College or AA degree	6,716	4,677	811	1,128	100	
Unknown	766	565	86	104	11	
BMI(mean ± ME)		27.58 ± 0.09	30.20 ± 0.16	31.40 ± 0.16	33.17 ± 0.53	<0.001
Normal:<25 kg/m^2^	7,517	6,264	597	614	42	
Overweight:25–30 kg/m^2^	7,480	5,186	954	1,256	84	
Obesity: ≥30 kg/m^2^	8,116	4,943	1,120	1964	189	
Unknown	276	176	33	58	9	
Smoking status						<0.001
Every day	3,989	2,849	487	611	42	
Some day	759	602	127	173	13	
Not at all	4,834	3,016	654	1,046	118	
Unknown	13,751	10,102	1,436	2062	151	
Alcohol status						***
Yes	86	39	17	29	1	<0.001
No	20,594	14,475	2,353	3,458	308	
Unknown	2,809	2055	334	405	15	
Hypertension						<0.001
Yes	6,956	4,092	931	1713	220	
No	16,463	12,428	1765	2,167	103	
Unknown	70	49	8	12	1	
Diabetes						<0.001
Yes	2,254	1,402	258	513	81	
Borderline	449	267	66	101	15	
No	20,770	14,887	2,379	3,276	228	
Unknown	16	13	1	2	0	

Multiple regression analysis results (Table 2) were established with different adjusting factors: Model one adjusted for gender, age, and race; Model two adjusted for gender, age, race, education level, BMI category, and smoking; Model three adjusted for gender, age, race, education level, BMI category, smoking, diabetes, and hypertension. In the fully adjusted model, there was no significant association between SUA and psoriasis (β = 0.999, 95% CI: 0.998–1.001, *p* = 0.275). Stratification by gender or race/ethnicity also revealed no significant relationship between SUA and psoriasis.

**TABLE 2:** Multivariable regression analysis of SUA and psoriasis.

	Model 1 β (95% CI), *p*-value	Model 2 β (95% CI), *p*-value	Model 3 β (95% CI), *p*-value
Serum uric acid (μmol/L)	1.001 (1.000, 1.002) 0.057	0.999 (0.998, 1.001) 0.508	0.999 (0.998, 1.001) 0.275
Subgroup analysis stratified by gender	*p* = 0.004	*p* = 0.321	*p* = 0.381
Male	β = 0.999 (0.998, 1.001)	β = 0.999 (0.996, 1.001)	β = 0.999 (0.996, 1.001)
	*p* = 0.342	*p* = 0.288	*p* = 0.251
Female	β = 1.003 (1.001, 1.005)	β = 1.000 (0.998, 1.003)	β = 1.000 (0.997, 1.002)
	*p* = 0.001	*p* = 0.851	*p* = 0.768
Subgroup analysis stratified by race/ethnicity	*p* = 0.084	*p* = 0.514	*p* = 0.516
Whites	β = 1.001 (1.000, 1.002)	β = 0.999 (0.997, 1.001)	β = 0.999 (0.997, 1.000)
	*p* = 0.179	*p* = 0.241	*p* = 0.135
Blacks	β = 1.002 (0.999, 1.005)	β = 1.004 (0.998, 1.010)	β = 1.003 (0.997, 1.009)
	*p* = 0.262	*p* = 0.224	*p* = 0.279
Other Hispanic	β = 1.004 (1.001, 1.007)	β = 1.003 (0.999, 1.008)	β = 1.003 (0.998, 1.008)
	*p* = 0.008	*p* = 0.164	*p* = 0.249
Other race/ethnicity	β = 1.001 (0.999, 1.004)	β = 1.000 (0.995, 1.005)	β = 0.999 (0.994, 1.004)
	*p* = 0.340	*p* = 0.948	*p* = 0.758

### 3.2 Results of mendelian randomization analysis

A total of 268 SNPs associated with uric acid levels were identified, with F statistics exceeding 10, indicating robust instrument strength (Xiang et al., 2021). We excluded palindromic SNPs with intermediate allele frequencies (Supplementary Table S2). Ultimately, 227 SNPs were retained for subsequent MR analysis. Detailed information regarding the instrumental variables can be found in Supplementary Tables S3, S4.

**FIGURE 3:**
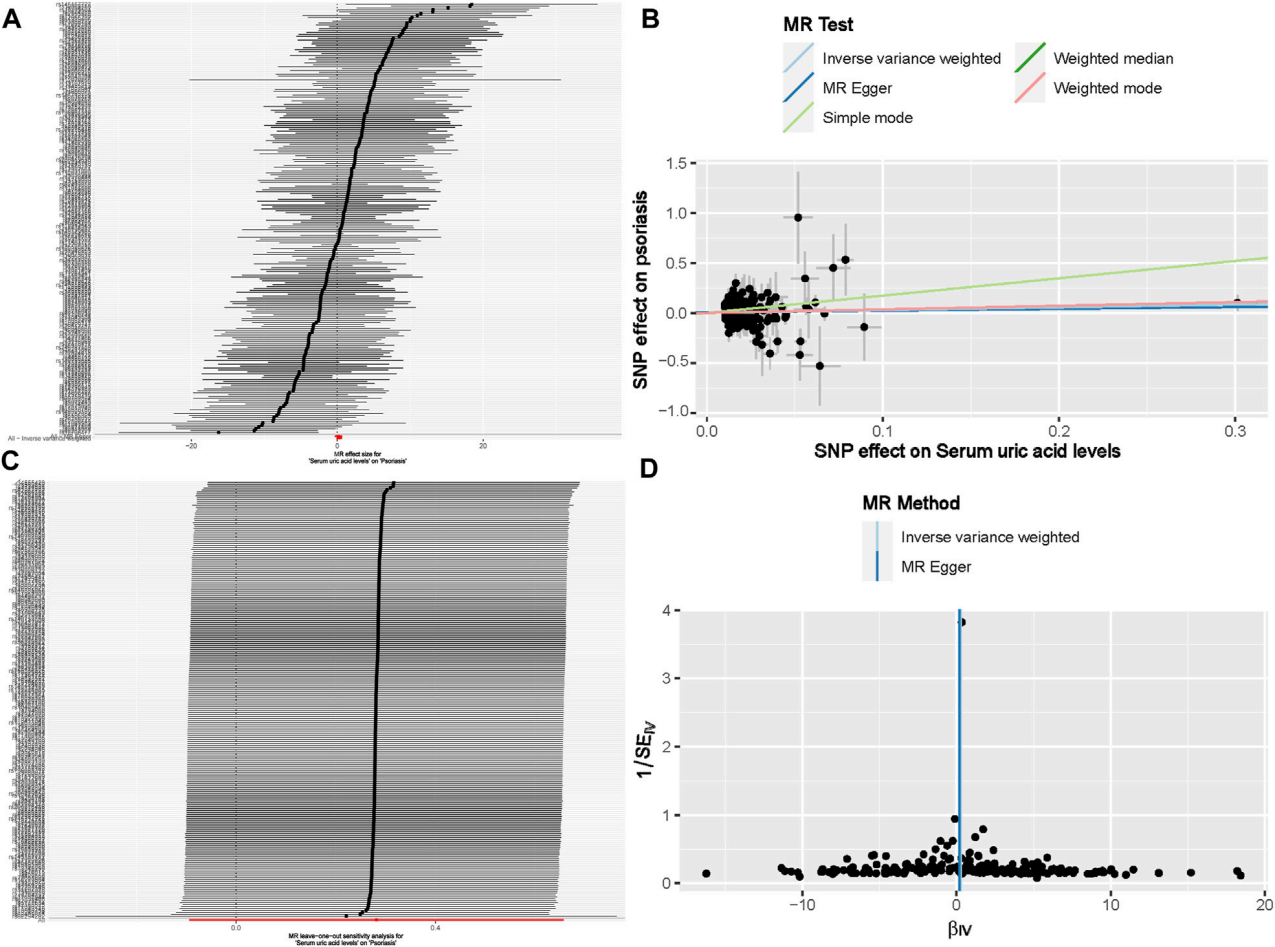
The causal effect of uric acid levels on psoriasis risk **(A)** Forest plot; **(B)** Scatter plot; **(C)** Sensitivity analysis; **(D)** Funnel plot. Note: For a complete list of SNP names, readers can refer to the appendix to obtain more detailed information.

**FIGURE 4:**
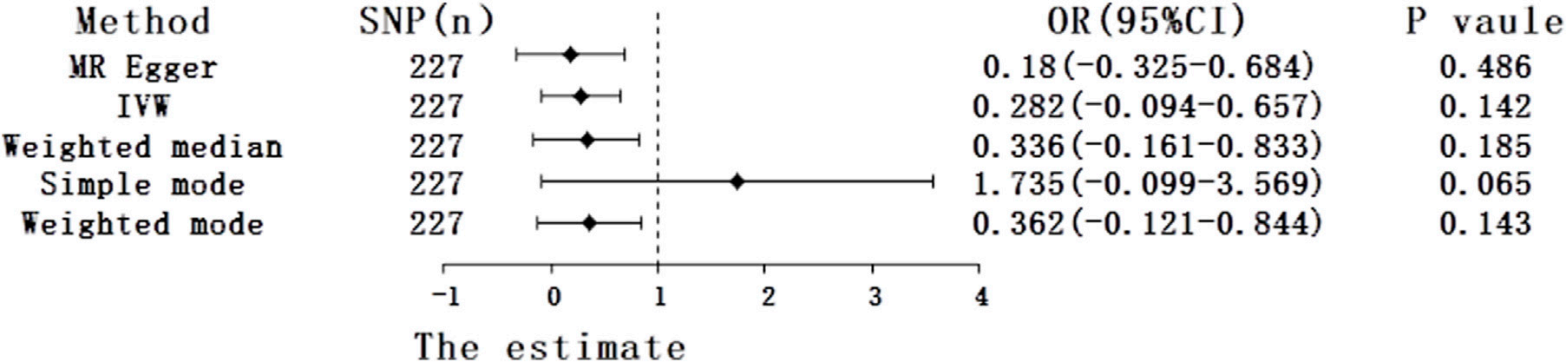
Forest plot of five Mendelian randomization estimators of the effect of uric acid levels on psoriasis.

Under the primary IVW method, the findings revealed no statistically significant causal link between uric acid levels and psoriasis (β = 0.281, 95% CI: -0.094–0.657, *p* = 0.142) (Figure 3A). Similarly, the results from the four other MR analysis methods failed to demonstrate any significant associations, thereby underscoring the absence of a causal relationship between elevated uric acid and the onset of psoriasis (Figure 4). Heterogeneity testing indicated a lack of heterogeneity in the IVW method (IVW: Q = 234.03, *p* = 0.342). Furthermore, the MR-Egger intercept test did not provide evidence of horizontal pleiotropy between instrumental variables and outcomes (IVW: *p* = 0.552) (Figure 3B). The leave-one-out sensitivity analysis confirmed that the MR analysis results were not significantly influenced by any individual SNP (Figure 3C). The funnel plot’s asymmetry also suggested the presence of heterogeneity (Figure 3D). Utilizing the MR-PRESSO method, two outliers (rs13230509, rs7773175) were identified, and upon their removal, the results remained unchanged (IVW: *p* = 0.084) (Table 3). The observational analysis indicated that there was no significant association between SUA and psoriasis, even after thorough adjustment for potential confounders. This finding was further supported by the MR analysis, which demonstrated a lack of causal relationship between elevated SUA levels and the onset of psoriasis. Additionally, the utilization of MR analysis, known for its ability to mitigate confounding and reverse causation biases, provided supplementary evidence that reinforces the conclusions drawn from the observational analysis. The consistency observed between the observational and MR analyses enhances the validity of the conclusion that SUA may not play a causal role in the development of psoriasis.

**TABLE 3:** MR results of causal links between serum uric acid and psoriasis risk.

Exposure	Outcome	SNPs	Methods	ß (95% CI)	*p*-Value	Horizontal pleiotropy MR-PRESSO				Heterogeneity			F statistic
						Egger intercept	SE	*p*-Value	Global test value	Cochran’s Q	*p*-Value	*p-value	
SUA	Psoriasis	227	MR Egger	0.18 (-0.325–0.684)	0.486	0.005	0.008	0.552	0.528	233.7	0.332	0.332	174.73
			IVW	0.282 (-0.094–0.657)	0.142					234.0	0.342	0.343	
			Weighted median	0.336 (-0.161–0.833)	0.185								
			Simple mode	1.735 (-0.099–3.569)	0.065								
			Weighted mode	0.362 (-0.121–0.844)	0.143								

IVW: inverse variance weighted; SUA: serum uric acid; *: The result of recalculation after removing outliers.

## 4 Discussion

In this study, we employed a combined approach of retrospective cross-sectional analysis and MR analysis. The conclusive findings from both methodologies consistently indicate the absence of a significant causal relationship between uric acid levels and psoriasis. Our approach stands out from previous studies as it combines an observational study with MR analysis. To the best of our knowledge, our study is the first to employ this integrated strategy to investigate the association between uric acid levels and psoriasis.

From a biological perspective, elevated uric acid levels can lead to the formation of uric acid crystals. These crystals, through various mechanisms, have the potential to activate keratinocytes and stimulate plasmacytoid dendritic cells to release pro-inflammatory cytokines such as IL-12 and IL-23. Consequently, this cascade of events induces the proliferation and differentiation of T cells and promotes the proliferation of keratinocytes, ultimately contributing to the development of psoriasis. Furthermore, individuals with psoriasis exhibit excessive proliferation of keratinocytes, resulting in an accelerated breakdown of nucleic acids and an increase in uric acid synthesis (Tripolino et al., 2021). Additionally, the release of cytokines, including IL-17, can contribute to non-alcoholic fatty liver disease, leading to decreased ATP production in the liver and consequently an increase in uric acid production (Huang et al., 2020). It is also plausible that hyperuricemia might be an outcome of the metabolic syndrome associated with psoriasis (Perez-Chada and Merola, 2020).

Our research findings exhibit a noticeable disparity with previous studies. Earlier observational studies attempted to unveil potential connections between psoriasis and hyperuricemia. Psoriasis has traditionally been regarded as a systemic disease, associated with various conditions, including metabolic syndrome. High serum uric acid levels have also been linked to components of metabolic syndrome (Lai et al., 2018). Consequently, several observational studies have endeavored to unveil the potential link between psoriasis and hyperuricemia. For instance, a cross-sectional study found a close association between hyperuricemia and psoriasis, as well as the Psoriasis Area and Severity Index (PASI) score, suggesting the monitoring of serum uric acid levels in psoriasis patients during treatment and follow-up (Yilmaz et al., 2017). Furthermore, a recent retrospective cohort study included 196 psoriasis patients and 191 age and gender-matched healthy controls. The results indicated significantly higher serum uric acid levels and a higher prevalence of hyperuricemia among psoriasis patients when compared to the healthy control group (Zhao et al., 2022). These findings appeared to support the role of uric acid in the onset and severity of psoriasis. Nevertheless, some studies have raised questions regarding these results, suggesting that the level of uric acid might not have a direct correlation with the extent of skin involvement. Instead, it might be related to metabolic changes associated with psoriatic arthritis (PsA), particularly high cholesterol levels and renal impairment (Bruce et al., 2000). Additionally, a study pointed out that in psoriasis patients in Hong Kong, there was no correlation between uric acid levels and the extent, or severity of skin lesions, blood lipid profiles, and kidney function (Lai et al., 2018). However, it is crucial to note that these studies are subject to variations in patient populations and the presence of multiple comorbidities, which currently generate a debate around the relationship (Kan et al., 2024). Therefore, comprehensive research with large sample sizes is required to thoroughly investigate the impact of uric acid levels on psoriasis.

This study harnesses data from NHANES and publicly accessible GWAS databases to amalgamate retrospective cross-sectional research with MR analysis. The primary aim is to elucidate whether uric acid levels contribute to an increased risk of psoriasis. The substantial sample size and methodological rigor in our study instill confidence in the conclusion that there appears to be no discernible causal relationship between uric acid levels and psoriasis.

Our observational investigation scrutinized the association between uric acid levels and the risk of psoriasis and found no significant correlation between uric acid levels and a heightened risk of psoriasis. Furthermore, in the context of MR analysis, employing five distinct methods including the IVW approach, the consistent outcome underscores the absence of a substantial correlation between uric acid concentration and the increased risk of psoriasis. As such, these results can be considered robust. It is worth noting that our cross-sectional study was conducted within the U.S. population, while the GWAS data used for MR analysis predominantly originated from European populations (Zhao et al., 2023). This, to a certain extent, suggests the generalizability of the results across diverse populations.

Our study boasts several strengths. Firstly, we ensured a sufficiently large sample size by utilizing the NHANES database. Secondly, we integrated observational research with MR analysis. Relying solely on the NHANES study might not permit causal inference, and MR analysis effectively addresses the limitations of observational studies, mitigating issues related to reverse causality and confounding factors. Additionally, the application of large-scale GWAS data in our MR analysis provided robust statistical power to assess the relationship between uric acid levels and psoriasis.

Nonetheless, our study is not without limitations. In our observational research, psoriasis information was gathered through questionnaires, introducing potential measurement bias. Furthermore, while Mendelian randomization methods improve control over confounding factors, they cannot entirely eliminate all potential sources of confounding. Thus, we cannot completely exclude the potential impact of these comorbidities on our results. Additionally, our study population primarily consisted of individuals from Europe and the United States, rendering our findings geographically limited. Lastly, The self-reported data within NHANES could also be susceptible to recall bias or influenced by social desirability biases.

For future research, addressing the limitations identified in our study will be crucial to deepen our understanding of the relationship between uric acid levels and psoriasis. Alternative research methodologies, such as machine learning algorithms or sophisticated statistical models, could be explored to capture the intricate relationships and potential interactions between uric acid and psoriasis more comprehensively. These advanced approaches might unveil patterns or insights that traditional methods might overlook.

In conclusion, our study employed a comprehensive approach to investigate the association between uric acid levels and psoriasis, offering novel insights. Although our results suggest that elevated uric acid is not a pronounced risk factor for psoriasis, further in-depth research is warranted in this field to unravel the additional intricacies of this relationship. Such endeavors hold significant implications for the treatment and prevention of psoriasis.

## 5 Conclusion

There is no significant causal relationship between uric acid levels and psoriasis. This finding provides valuable insights for the treatment and prevention of psoriasis, indicating that solely reducing uric acid levels may not be an effective strategy to reduce the risk of developing psoriasis. Future research should continue to explore other potential factors that may influence the onset of psoriasis, aiming for a better understanding and management of this chronic condition.

## Data Availability

The original contributions presented in the study are included in the article/Supplementary Material, further inquiries can be directed to the corresponding authors.
