# Supplementary material for: The impact of serum uric acid on psoriasis: NHANES 2005–2014 and Mendelian randomization
Source: Front Genet. 2024 May 9;15:1334781. doi: 10.3389/fgene.2024.1334781 (PMC11111913; doi:10.3389/fgene.2024.1334781)
Supplement: Supplementary file 1 [file Table1.DOCX]

TABLE 1 Description of GWAS summary statistics.

| Phenotype | Year | Author | Ancestry | Sample size | Number of SNPs | PMID | Data sources |
| --- | --- | --- | --- | --- | --- | --- | --- |
| SUA | 2022 | Sakaue S | European | 343,836 | 19,041,286 | 34594039 | ebi-a-GCST90018977 |
| Psoriasis | 2021 | Mbatchou J | European | 407,746 | 11,039,125 | 34017140 | ebi-a-GCST90013885 |

SUA:serum uric acid
