# Supplementary material for: The impact of serum uric acid on psoriasis: NHANES 2005–2014 and Mendelian randomization
Source: Front Genet. 2024 May 9;15:1334781. doi: 10.3389/fgene.2024.1334781 (PMC11111913; doi:10.3389/fgene.2024.1334781)
Supplement: Supplementary file 2 [file Table2.DOCX]

Harmonising Serum uric acid levels || id:ebi-a-GCST90018977 (ebi-a-GCST90018977) and Psoriasis (Firth correction) || id:ebi-a-GCST90013885 (ebi-a-GCST90013885)

Removing the following SNPs for incompatible alleles:

rs34555420

Removing the following SNPs for being palindromic with intermediate allele frequencies:

rs10771025, rs10901057, rs11164916, rs114158982, rs114165349, rs12510175, rs12543287, rs12775853, rs12891886, rs12911430, rs13021972, rs13230509, rs13418518, rs144188351, rs150147865, rs17632159, rs1851285, rs1869581, rs187355703, rs1925258, rs2252862, rs2493121, rs2644128, rs4476815, rs45499402, rs523288, rs538737, rs59864860, rs6825697, rs7039, rs703978, rs7310615, rs738409, rs75523587, rs7773175, rs78671965, rs831036, rs9534949, rs9927317
